# Overexpression of the HIF Hydroxylases PHD1, PHD2, PHD3 and FIH Are Individually and Collectively Unfavorable Prognosticators for NSCLC Survival

**DOI:** 10.1371/journal.pone.0023847

**Published:** 2011-08-22

**Authors:** Sigve Andersen, Tom Donnem, Helge Stenvold, Samer Al-Saad, Khalid Al-Shibli, Lill-Tove Busund, Roy M. Bremnes

**Affiliations:** 1 Institute of Clinical Medicine, University of Tromso, Tromso, Norway; 2 Department of Oncology, University Hospital of North Norway, Tromso, Norway; 3 Institute of Medical Biology, University of Tromso, Tromso, Norway; 4 Department of Pathology, University Hospital of North Norway, Tromso, Norway; 5 Department of Pathology, Nordland Central Hospital, Bodo, Norway; Sun Yat-sen University Cancer Center, China

## Abstract

**Introduction:**

Hypoxia induced factors (HIFs) are at the heart of the adaptive mechanisms cancer cells must implement for survival. HIFs are regulated by four hydroxylases; Prolyl hydroxylase (PHD)-1,-2,-3 and factor inhibiting HIF (FIH). We aimed to investigate the prognostic impact of these oxygen sensors in NSCLC.

**Methods:**

Tumor tissue samples from 335 resected stages I to IIIA NSCLC patients was obtained and tissue microarrays (TMAs) were constructed. Hydroxylase expression was evaluated by immunohistochemistry.

**Principal Findings:**

There was scorable expression for all HIF hydroxylases in tumor cells, but not in stroma. In univariate analyses, high tumor cell expression of all the HIF hydroxylases were unfavorable prognosticators for disease-specific survival (DSS); PHD1 (P = 0.023), PHD2 (P = 0.013), PHD3 (P = 0.018) and FIH (P = 0.033). In the multivariate analyses we found high tumor cell expression of PHD2 (HR = 2.03, CI 95% 1.20–3.42, P = 0.008) and PHD1 (HR = 1.45, CI 95% 1.01–2.10, P = 0.047) to be significant independent prognosticators for DSS. Besides, there was an additive prognostic effect by the increasing number of highly expressed HIF hydroxylases. Provided none high expression HIF hydroxylases, the 5-year survival was 80% vs. 23% if all four were highly expressed (HR = 6.48, CI 95% 2.23–18.8, P = 0.001).

**Conclusions:**

HIF hydroxylases are, in general, poor prognosticators for NSCLC survival. PHD1 and PHD2 are independent negative prognostic factors in NSCLC. Moreover, there is an additive poor prognostic impact by an increasing number of highly expressed HIF hydroxylases.

## Introduction

Due to its high prevalence and poor survival, lung cancer is the leading cause of cancer-related deaths [1]. Eighty to 85% of lung cancers are of non-small cell type (NSCLC). At early stages NSCLC is potentially curable by surgery [2], but even among tumor-resected patients lung cancer mortality remains high. There is a need for better prognostic and predictive factors, incorporated with clinicopathological features, for treatment stratification, as well as new treatment options [2].

Hypoxia is a feature of many NSCLC tumors [3] and the ability of tumor cells to adapt to a reduced oxygen and nutrient supply is vital for their survival [4]. When oxygen tension is reduced, the HIF transcription factors, composed of the subunits HIFα (HIF1α, HIF2α or HIF3α) and HIFβ, are at the heart of these mechanisms. They control the cellular expression of hundreds of target genes, which makes the tumor cell capable of surviving in a hypoxic microenvironment [5]. Regulation of the HIF activity is mainly controlled by the half-life of the HIFα-subunit, which is tightly controlled by the oxygen dependent hydroxylation by HIF hydroxylases. Under normoxia, HIFα is hydroxylated by prolyl hydroxylases (PHD1, PHD2 and PHD3) and factor inhibiting HIF (FIH). Hydroxylation through PHDs enables binding with von Hippel-Lindau (VHL) tumor suppression protein with subsequent targeting of HIFα for proteosomal degradation by ubiquitation [6], [7]. All PHDs have the same function, but appears to have different specificities for various hydroxylation sites [8]. PHD2 is the most abundant form and the main regulator of HIF1 activity, whereas PHD3 more efficiently regulates HIF2α [8], [9]. Together with the transcriptional modifyer FIH, these are known as HIF hydroxylases. These serve the function as oxygen sensors in the vital cellular oxygen homeostasis [8], [10].

Although HIF hydroxylases recently were recognized as important players in cancer biology by interfering with angiogenesis and metastasis [11], the role of these oxygen sensors in tumorigenesis is poorly defined. They have been proposed as both tumor suppressors and drivers of tumorigenesis [12]. Antibodies for detection of these proteins in paraffin-embedded human tissues have recently been developed and validated [13]. Only one previous study has evaluated these HIF hydroxylases in NSCLC tumors, but without assessing their prognostic relevance [14].

We aimed to pioneer the first comprehensive prognostic impact evaluation of the HIF hydroxylases in a large unselected NSCLC cohort. Studies evaluating the clinical significance of these markers in malignancy are limited, and they have a potential role as therapeutic targets [12].

## Results

### Patient characteristics

The patients' demographic, clinical and histopathological data are presented in Table 1. The median follow-up time of survivors was 86 months (range 48–216). The median patient age was 67 (range 28–85), 75% were male, 95% had performance status 0–1 and 95% were present or previous smokers. The NSCLC tumors comprised 191 squamous cell carcinomas (SCC), 113 adenocarcinomas (AC) including 18 bronchioalveolar carcinomas (BAC), and 31 large-cell carcinomas (LCC).

**Table 1:** Patient characteristics and clinicopathological variables and their prognostic value for disease-specific survival in 335 NSCLC patients (univariate analyses; log rank test).

Characteristic	Patients(n)	Patients(%)	Median survival(months)	5-Year survival(%)	P
**Age**					
≤65 years	156	47	83	55	0.34
>65 years	179	53	NR	60	
**Sex**					
Female	82	25	190	63	0.20
Male	253	75	83	56	
**Smoking**					
Never	15	5	19	43	0.23
Current	215	64	NR	60	
Former	105	31	71	54	
**Performance status**					
PS 0	197	59	NR	63	**0.013**
PS 1	120	36	64	52	
PS 2	18	5	25	33	
**Weight loss**					
<10%	303	90	127	58	0.71
>10%	32	10	98	57	
**Histology**					
SCC	191	57	NR	66	0.08
AC	113	34	54	45	
LCC	31	9	98	56	
**Differentiation**					
Poor	138	41	47	47	**<0.001**
Moderate	144	43	190	64	
Well	53	16	NR	68	
**Surgical procedure**					
Lobectomy+Wedge*	243	73	190	61	**0.004**
Pneumonectomy	92	27	37	47	
**Pathological stage**					
I	157	47	190	71	**<0.001**
II	136	40	61	51	
IIIa	42	13	17	23	
**Tumor status**					**<0.001**
1	85	25	190	74	
2	188	56	84	57	
3	62	19	25	36	
**Nodal status**					
0	232	69	190	66	**<0.001**
1	76	23	35	43	
2	27	8	18	18	
**Surgical margins**					
Free	307	92	190	58	0.29
Not free	28	8	47	47	
**Vascular infiltration**					
No	284	85	190	58	**<0.001**
Yes	51	15	27	32	

*Wedge, n = 10.

Abbreviations: NR = not reached; PS = Performance status; SCC = Squamous cell carcinoma, LCC = Large-cell carcinoma; AC = Adenocarcinoma (including bronchioloalveolar carcinoma).

### Expression of HIF hydroxylases

All HIF hydroxylases were detectable for scoring in NSCLC tumor cells. In the surrounding tumor stroma some expression was seen, especially in endothelial cells. However, in our TMA-sections it was not scorable. The localization of staining in tumor cells was predominantly cytoplasmic although there was some nuclear staining for all antibodies. Nuclear staining, however, was almost always accompanied by a strong cytoplasmic staining and the scarce number of cores with exclusive nuclear staining was not statistically evaluable for prognosis. A dominant intensity (>50% of viable tumor cells) was evaluable for all tumor cores with only minor heterogeneity within cores. PHD3 expression in BACs was the only exception, where a more pronounced heterogeneity was observed with some tumor cells strongly expressing PHD3.

The expression in normal lung and surrounding stroma was fairly similar to the findings by Giatromanolaki et al [14] except for the stronger granulated positivity in peritumoral pneumocytes compared to pneumocytes in normal lung.

### Correlations

There were no correlations between HIF hydroxylases and the clinicopathological variables in Table 1, but weak correlations between various HIF hydroxylases were observed; PHD1 vs. PHD2 (r = 0.107, P = 0.057), vs. PHD3 (r = 0.079, P = 0.16) and vs. FIH (r = 0.14, P = 0.013); PHD2 vs. PHD3 (r = 0.083, P = 0.137) and PHD2 vs. FIH (r = 0.183, P = 0.001); PHD3 vs. FIH (r = 0.051, P = 0.363).

When the HIF hydroxylases were compared with tumor cell expression of molecular markers previously published by this group [15]–[19], the following correlations were observed; PHD1 vs. VEGF-A (r = 0.27, P = <0.001) and PDGF-A (r = 0.23, P<0.001); PHD2 vs. VEGFR3 (r = 0.23, P<0.001); FIH vs. VEGFR3 (r = 0.25, P<0.001), Notch4 (r = 0.26, P<0.001), HIF2α (r = 0.22, P<0.001), LDH5 (r = 0.22, P<0.001), Ang-1 (r = 0.21, P<0.001), Ang-4 (r = 0.24, P<0.001), and Tie-2 (r = 0.22, P<0.001).

### Univariate analyses

Results regarding the clinicopathological variables are presented in Table 1. WHO performance status (P = 0.013), differentiation (P<0.001), surgical procedure (P = 0.004), pathological stage (P<0.001), T-status (P<0.001), N-status (P<0.001) and vascular infiltration (P<0.001) were significant prognostic factors (Table 1).

**Figure 1:**
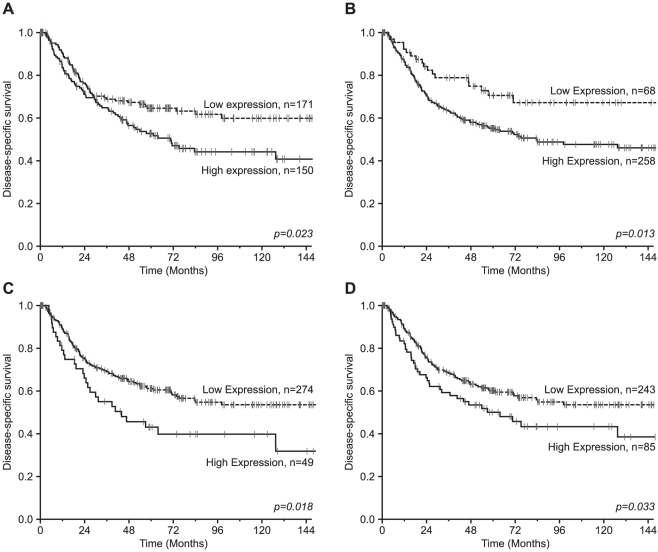
Disease-specific survival according to HIF hydroxylase expression. Disease-specific Kaplan-Meier survival curves according to: A) PHD1, B) PHD2, C) PHD3 and D) FIH in resected NSCLC patients. The P-value is according to the log-rank test.

Data on the association between molecular markers and DSS are given in Table 2 and Figure 1. For all of the HIF hydroxylases, high tumor cell expression was significantly associated with poor survival; PHD1 (P = 0.023), PHD2 (P = 0.013), PHD3 (P = 0.018) and FIH (P = 0.033).

**Table 2:** Expression of oxygen sensors in tumors as prognostic factors for disease-specific survival in 335 NSCLC patients (univariate analyses; log-rank test).

Characteristics	Patients(n)	Patients (%)	Median survival (months)	5-year survival (%)	*P*
PHD1					0.023
**High**	**150**	**45**	**71**	**53**	
**Low**	**171**	**51**	**190**	**65**	
**Missing**	**14**	**4**			
PHD2					0.013
**High**	**258**	**77**	**83**	**55**	
**Low**	**68**	**20**	**NR**	**71**	
**Missing**	**9**	**3**			
PHD3					0.018
**High**	**49**	**15**	**44**	**43**	
**Low**	**274**	**82**	**190**	**61**	
**Missing**	**12**	**3**			
FIH					0.033
**High**	**85**	**25**	**64**	**50**	
**Low**	**243**	**73**	**NR**	**60**	
**Missing**	**7**	**2**			
Co-expression of oxygen sensors					<0.001
**0**	**36**	**11**	**NR**	**80**	
**1**	**106**	**32**	**NR**	**65**	
**2**	**112**	**33**	**190**	**57**	
**3**	**52**	**15**	**57**	**48**	
**4**	**10**	**3**	**24**	**23**	
**Missing**	**19**	**6**			

**Figure 2:**
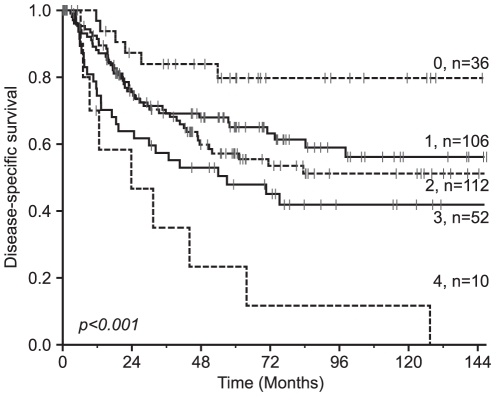
Disease-specific survival according to the co-expression of HIF hydroxylases. Disease-specific Kaplan-Meier survival curves according to the sum of HIF hydroxylases with a high expression in resected NSCLC patients. The P-value is according to the log-rank test.

When assessing the co-expression variable of all the HIF hydroxylases, there was a significant additive pattern with a progressively worse survival with the increasing number (0–4) of highly expressed HIF hydroxylases (Table 2 and Figure 2).

### Multivariate analyses

Results of the multivariate analyses are presented in Table 3. In Model 1 we found high tumor cell expression of the PHD2 (HR = 2.03, CI 95% 1.20–3.42, P = 0.008) and PHD1 (HR = 1.45, CI 95% 1.01–2.10, P = 0.047) to be significant independent prognosticators for DSS in addition to several clinicopathological variables (tumor status, P<0.001; nodal status, P<0.001; performance status, P = 0.001; vascular infiltration; P = 0.002; differentiation, P = 0.006). High tumor cell expression of PHD3 (P = 0.058) and FIH (P = 0.15) did not, however, reach statistical significance.

**Table 3:** Results of Cox regression analyses (Backward stepwise model).

	Model 1(all significant variables entered)			Model 2(Clinicopathological and co-expression variable entered)		
Factor	Hazard Ratio	95% CI	P	Hazard Ratio	95% CI	P
**Tumor status**			**<0.001** *			**<0.001** *
1	1			1		
2	1.72	1.02–2.89	**0.041**	1.95	1.14–3.36	**0.015**
3	3.27	1.84–5.81	**<0.001**	3.72	2.03–6.85	**<0.001**
**Nodal status**			**<0.001** *			**<0.001** *
0	1			1		
1	1.92	1.25–2.94	**0.003**	1.94	1.26–2.97	**0.002**
2	3.30	1.95–5.59	**<0.001**	3.03	1.75–5.23	**<0.001**
**Performance status**			**0.001** *			**0.001** *
ECOG 0	1			1		
ECOG 1	2.02	1.37–2.98	**<0.001**	2.03	1.37–2.99	**<0.001**
ECOG 2	2.16	0.95–4.91	0.065	2.10	0.92–4.77	0.077
**Vascular infiltration**			**0.002**			**0.002**
No	1			1		
Yes	2.08	1.29–3.34		2.13	1.31–3.49	
**Differentiation**			**0.006** *			**0.006** *
Well	1			1		
Moderate	1.89	0.97–3.69	0.061	1.62	0.83–3.14	0.16
Poor	1.02	0.51–2.02	0.096	0.85	0.42–1.72	0.65
**PHD1**			**0.047**			
Low	1					
High	1.45	1.01–2.10				
**PHD2**			**0.008**			
Low	1					
High	2.03	1.20–3.42				
**Co-expression** †						**<0.001** *
0				1		
1				1.95	0.81–4.66	0.135
2				2.05	0.86–4.89	0.105
3				4.13	1.69–10.1	**0.002**
4				6.48	2.23–18.8	**0.001**

*Overall significance as a prognostic factor.

† The co-expression variable, included all of the four oxygen sensors and was stratified by the total number of oxygen sensors with a high expression.

Grey boxes indicate variables not entered in the analysis.

In model 2, we found a gradually increasing hazard ratio for lung cancer death in patients with an increasing number of highly expressed HIF hydroxylases in comparison to those with no high expression of hydroxylases (Table 3). Patients with high tumor cell expression of all four HIF hydroxylases had a HR of 6.48 (CI 2.23–18.8, P = 0.001).

## Discussion

We present the first large-scale study evaluating the prognostic impact of HIF hydroxylases in surgically resected NSCLC. Using validated antibodies, in this large unselected cohort, we found that high expression of all the HIF hydroxylases were prognosticators for poor survival, with PHD1 and especially PHD2 as independent negative prognostic factors. In addition, there was an additive poor prognostic impact by the increasing number (0–4) of highly expressed HIF hydroxylases.

A limited number studies have evaluated the expression of HIF hydroxylases in various cancers [13], [20]–[27] including one in NSCLC [14]. Only three have assessed survival outcome in relation to expression of these HIF hydroxylases [21]–[23], none in NSCLC. In general, IHC-studies have found increased, but variable staining of PHDs and FIH in human cancers [12]. Corroborating previous studies [21], [22], [24], [25], [27], we did not find the expected simplistic association between high HIF hydroxylase expression and low HIFα expression, or vice versa, as expected from earlier functional studies [12]. Consistent with Giatromanolaki et al [14], the most significant, though weak, correlations were between PHD1-FIH and PHD2-FIH. Expression patterns were also similar, but in our TMA cores there were no clear examples of nuclear expression without an accompanying strong cytoplasmic expression.

In pancreatobiliary ampullary adenocarcinoma, Gossage et al. observed that high PHD3 expression was significantly associated to a worse overall survival [23]. There was also a similar trend for PHD2. In pancreatic endocrine tumors, Couvelard and colleagues found high nuclear staining of PHD1 and PHD3 and stromal staining of FIH to correlate with a worse survival [22]. In prostate cancer, Boddy et al. did not observe any associations between PHDs and survival or PSA recurrence [21]. To summarize, few small studies have examined possible associations between HIF hydroxylase expression and clinical outcome, and the positive ones have revealed an association with reduced survival.

The earlier functional studies on HIF hydroxylases identified them as downregulators of HIFα. The fact that high expression of HIF hydroxylases serve as poor prognosticators for DSS seemingly contradicts the canonical function as inhibitors of HIFα, which has consistently been shown to be a tumor progression marker [5]. Several experimental studies have recently tried to elucidate essential roles of HIF hydroxylases in tumor progression. In line with our data, Henze et al. found that inhibition of PHDs significantly reduced glioblastoma cell survival and that PHD inhibition increased hypoxic cell death as well as death induced by chemotherapeutics [24]. Furthermore, the PHD/HIF regulatory axis was postulated as a novel therapeutic target to disable a tumor's ability to adjust to hypoxic conditions and maintain cell survival [24].

Mazzone et al did functional studies of the stromal role of PHD2 in tumorigenesis by implanting pancreatic tumors in immunocompromized PHD2^+/−^ mice [28]. Surprisingly, the heterozygous deficiency of PHD2 led to improved endothelial lining, vessel maturation, tumor perfusion and oxygenation with a subsequent inhibition of tumor cell invasion, intravasation and metastasis. The experimentally reduced available level of PHD2 in the host, actually seemed to reduce the malignancy of implanted tumors. Besides, Ginouves et al. found that chronic hypoxia (24 h to 7 days) increased the pool of PHDs and overactivated all three isoforms thereby “desensitizing” HIFα and protected cells from necrosis [29]. Desensitizing HIFα proved to be required since all experimental cells died if HIF1α expression was not reduced during chronic hypoxia. Using implanted colon carcinoma tumors with decreased PHD2 expression in mice, Chan et al observed that tumors grew dramatically faster than control tumors and that PHD2 loss also induced angiogenesis and recruitment of bone marrow-derived cells. [30]. In pancreatic cancer, Su et al. recently reported that PHD3 overexpression mediated tumor cell growth and invasion [26]. Overexpression of PHD1 was shown by Erez et al. to inhibit tumor growth [31].

In light of these functional studies on HIF hydroxylases in cancer, it is difficult to decipher why elevated expression levels of HIF hydroxylases are associated with a poor survival. The studies so far do not give us a clear functional explanation of the function of HIF hydroxylases in cancer. To quote Jokilehto and Jaakkola in a recent review, “*given the uncertainties in specific PHD function, their role in cancer is inconclusive at the best*” [12].

HIF hydroxylases appear to be important players in tumor biology. As high cellular levels of HIF hydroxylases seem to be important in the malignant phenotype, they may qualify as potential therapeutic targets in NSCLC. Due to the basic understanding of HIF hydroxylase functions, Nagel et al. recently proposed inhibition of HIFα through activation of PHDs. However, if PHDs are vital in disease progression, in consistency with our findings, these oxygen sensors would rather be a target of inhibition. [32]. Although several inhibitors of HIF hydroxylases are known, there are presently no ongoing studies in cancers registered on www.clinicaltrials.com [32].

In conclusion, we found high expression of all four HIF hydroxylases to be indicators of poor prognosis in NSCLC with PHD2 as the most significant prognostic marker. In addition, patients could be stratified in highly diverging prognostic subgroups according to the additive number of highly expressed HIF hydroxylases. We suggest HIF hydroxylases as possible molecular markers for prognostic stratification in addition to already incorporated clinicopathological prognosticators. We also propose them as potential targets for cancer growth inhibition.

## Materials and Methods

### Patients and Tissues

Retrospectively, we identified 371 patients who were surgically tumor-resected with pathological stage I to IIIA NSCLC at the University Hospital of North Norway and Nordland Central Hospital between 1990 and 2004. Primary tumor tissue was collected from the archives of the two pathology departments. After the necessary exclusion of 36 patients due to inadequate paraffin-embedded fixed tissue blocks (n = 13), other malignancy within the 5 years prior to diagnosis (n = 13) or having received radiotherapy or chemotherapy prior to surgery (n = 10), we were left with 335 eligible patients with complete demographic and clinicopathological data. The pathological data were revised according to the 7th edition of UICC TNM classification of lung cancer [33]. Adjuvant chemotherapy was not introduced in Norway in this period (1990–2004). The last disease-specific survival (DSS) update was done in November 2008. The Norwegian Data Inspection Board and The Regional Committee for research ethics have approved the study.

### Microarray construction

Duplicate 0.6 mm core biopsies from the most representative areas of tumor cells (neoplastic epithelial cells) and tumor stroma were collected from each surgical specimen using a tissue-arraying instrument (Beecher Instruments, Silver Springs, MD,USA). Normal lung tissue localized distant from the primary tumor as well as lung tissue samples from 20 patients without any history of malignancy were also sampled. Eight tissue microarray blocks (TMAs) were constructed to include all the cores. The detailed methodology has been reported previously [16], [17].

### Immunohistochemistry

Antibodies developed and published by the Nuffield Department of Clinical Laboratory Sciences at the University of Oxford, UK were donated [13], [14]. These were anti-PHD1 (PHD112, mouse monoclonal), anti-PHD2 (366G/76, mouse monoclonal, not diluted), anti-PHD3 (EG188e, mouse monoclonal, not diluted) and anti-FIH (162c/D6, mouse monoclonal, 1∶5). Probably due to low antibody concentration against PHD1 when experiments were done, we did not succeed in attaining a strong enough staining with the donated PHD1 antibody. The PHD-1 antibody in this study was therefore acquired from Abcam (ab82884, PHD112/G7, mouse monoclonal, 1∶50). The 4 µm TMA sections containing tissue cores were deparaffinized with xylene and rehydrated with ethanol before being subjected to the antibodies.

Negative controls were simultaneously performed for all antibodies by omitting the primary antibody. For the commercially antibody for PHD1 we used normal testis as a positive control. For the donated antibodies, staining patterns in tumor and normal lung were compared to what has been published earlier regarding staining with these antibodies in NSCLC and lung tissues to ensure the proper staining with these antibodies [13], [14]. Validation on transfected cell lines with positive and negative controls has previously been published [13], [14].

Antigen retrieval was done manually for all antibodies except PHD1. For PHD2 and FIH, antigen retrieval was done by placing the specimens in 0.01 M citrate buffer at pH 6.0 and exposed to microwave heating of 20 minutes at 450 W. The antigen retrieval for PHD3 was the same except for the buffer which was a 10 mom Tris/1 mM EDTA buffer at pH 9.0. All donated antibodies were incubated at room temperature (≈20°C) overnight except for FIH where the primary antibody was incubated for 30 min in room temperature. The methods were adapted from the donators. For PHD1 we used the Ventana Benchmark XT® (Ventana Medical Systems Inc.;Illkirch, France), procedure ultraview DAB v3 with automatic antigen retrieval with CC1 mild (30 min). Finally, all slides were counterstained with hematoxylin to visualize the nuclei.

### Scoring of immunohistochemistry

**Figure 3:**
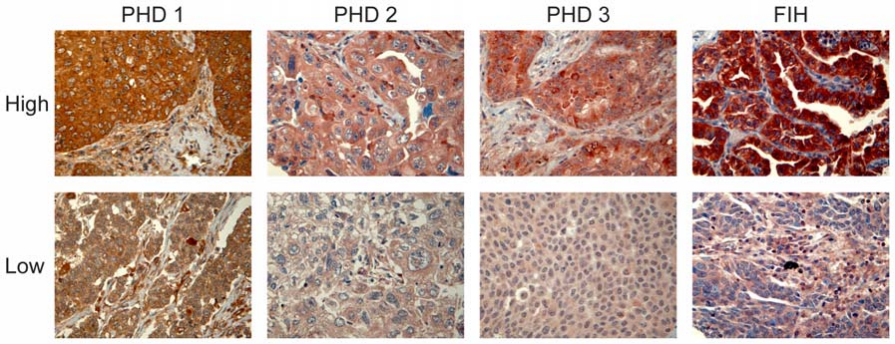
Immunohistochemical staining. Immunohistochemical analyses of NSCLC representing high and low intensities for tumor cell expression of PHD1, PHD2, PHD3 and FIH.

Viable parts of each anonymized core were scored independently and semiquantitatively by two pathologists (S.A.S and K.A.S) by light microscopy. When assessing a variable in a given core, the pathologists were blinded to the outcome and score of the other observer. Only the neoplastic cell compartment (tumor cells) was scored in this study as there was no scorable expression in the surrounding tumor stroma (stromal cells). The dominant staining intensity in tumor was scored as: 0 = negative, 1 = weak, 2 = intermediate or 3 = strong (Figure 3). Only cytoplasmic staining was scored. Interindividual variability with respect to IHC-scoring was evaluated on the current material in a previous paper (r = 0.95, range 0.93–0.98) [17].

A mean score was calculated for the two tumor cell cores in each individual. In tumor, high expression was defined as  = 3 for PHD1, PHD3 and FIH, and ≥2.0 for PHD2. Similar scoring methods have been used in our previous IHC-scoring studies [17], [34], [35] and by others [36]. Optimal statistical cut-off levels for high and low expression were used.

### Statistical methods

The statistical analyses were done using the SPSS 17.0.0 package (Chicago, IL). The χ2 test and Fishers exact tests were used to examine the associations between molecular marker expressions and the clinicopathological markers. *r*-values are the Spearman's rank correlation coefficient. Plots of the DSS, according to marker expressions, were drawn using the Kaplan-Meier method, and the statistical significance between survival curves was assessed by the log rank test. The survival curves were terminated at 146 months, due to less than 10% of patients at risk after this point. The chosen endpoint, DSS, was calculated from the time of surgery to the time of lung cancer death.

In the first model of the multivariate analysis (Model 1), all significant variables from the univariate analyses (except surgical procedure and pathological stage) were entered in a backward stepwise Cox regression analysis with a probability for stepwise entry and removal at 0.05 and 0.10, respectively. A P<0.05 was considered statistically significant for all analyses. In the second model for multivariate analysis (Model 2), all the significant clinicopathological variables (except surgical procedure and pathological stage) were entered as well as the co-expression variable. The co-expression variable, including all of the four HIF hydroxylases, was stratified by the number of HIF hydroxylases demonstrating high expression.

### Ethics statement

The Norwegian Data Inspection Board and The Regional Committee for research ethics have approved the study. Information and subsequent written consent from patients was considered, but as this was a retrospective study with more than half of patients deceased, the rest of the patients having to reminded about the death rate of the disease and the possible raising of unrealistic hope for the individual, they specifically waived the need for consent.
